# Editorial: Environmental exposomics and metabolic disorders

**DOI:** 10.3389/fendo.2023.1245239

**Published:** 2023-10-04

**Authors:** Luana Ricci Paulesu, Siyu Chen, Cristina de Angelis, Caixia Guo, Ning Shen

**Affiliations:** ^1^ Department of Life Sciences, University of Siena, Siena, Italy; ^2^ State Key Laboratory of Natural Medicines, China Pharmaceutical University, Nanjing, China; ^3^ Dipartimento di Medicina Clinica e Chirurgia, Sezione di Endocrinologia, Diabetologia ed Andrologia, Unità di Andrologia e Medicina della Riproduzione, Sessualità e Affermazione di Genere (FERTISEXCARES), Università Federico II di Napoli, Naples, Italy; ^4^ Department of Occupational Health and Environmental Health, School of Public Health, Capital Medical University, Beijing, China; ^5^ China Exposomics Institute, Nantong 4th hospital affiliated to Nanjing Medical University, Nanjing, China

**Keywords:** environmental contaminants, human health, human reproduction, sex hormone, endocrine disruption

Environmental contaminants burden is becoming increasingly serious, by raising concerns on the health effects of exposures to such compounds and by threatening society’s long-term growth (1); humanity has therefore an unprecedented task in achieving a sustainable global environment. Metabolic diseases are caused by a mix of hereditary variables and represent an incipient newly discovered target of environmental exposures (e.g., chemical, physical, and biological factors) (2, 3); moreover, sex hormones, acting as highly active metabolic hormones, have been long time identified as a major target of endocrine disruption by environmental contaminants (4). Extensive efforts have been engaged in unravelling the concrete, realistic, impact of environmental contaminants on metabolic health, leading to production of compelling data, particularly in experimental models; the study of the underlying molecular mechanisms that are involved in the pathogenesis of metabolic disorders could be indeed fundamental to preventing and attenuating the consequences for human health. This Research Topic focused mainly on the dissertation of the connections between external environmental factors and metabolic diseases in humans. In the original article by Xu et al., the effects of exposure to mercury, manganese, lead, cadmium, and selenium in adults from the general population were analyzed based on the National Health and Nutrition Examination Survey (NHANES). Authors showed that blood metal mixtures were positively associated with gout-related outcomes, with lead displaying the greatest effect on hyperuricemia and gout. The impact of exposure to environmental contaminants on human fertility is also discussed in the original article from Yuan et al., focused on polycyclic aromatic hydrocarbons (PAHs), a family of environmental toxicants produced mainly by incomplete combustion of organic compounds, including diesel, gasoline, coal, oil, and wood. PAHs can be inhaled through smoking, vehicle exhaust and industrial emissions and ingested through contaminated food. This study showed disrupting effects of PAH exposure on the sex hormones testosterone and estradiol levels in male and female adults. The relationship between environmental contaminants exposure and impaired sex hormones homeostasis was further investigated in the original article by Wei et al. aimed to analyze the association between Volatile organic compounds (VOCs) and sex hormones using multiple statistical models. Authors reported that exposure to VOCs might be associated with sex hormone metabolic disorder in American adult males. Exogenous factors associated with the development of metabolic syndrome (MetS) were studied by Pei et al. investigating the effects of multiple water-soluble vitamins, such as vitamin C, vitamin B9, and vitamin B12 on the occurrence of MetS. Employing the NHANES 2003-2006 and using logistic regression models, the authors revealed that high serum levels of water-soluble vitamins were associated with a reduced MetS risk.

## Author contributions

All authors listed have made a substantial, direct, and intellectual contribution to the work and approved it for publication.
